# Supplementary material for: Altered microRNA Transcriptome in Cultured Human Liver Cells upon Infection with Ebola Virus
Source: Int J Mol Sci. 2021 Apr 6;22(7):3792. doi: 10.3390/ijms22073792 (PMC8038836; doi:10.3390/ijms22073792)
Supplement: Supplementary file 1 [file ijms-22-03792-s001.zip › Supplementary_File/C_ GO_Analysis_Results/16-30nt_go_Mayinga-24h-Huh7_vs_Control-24h-Huh7_down.mature_mirna_targets/CC_result(Human).html]

| GO.ID | Term | Ontology | Count | Pop.Hits | List.Total | Pop.Total | Fold.Enrichment | Pvalue | FDR | Enrichment.Score | Gene.Ratio | GENES |
| --- | --- | --- | --- | --- | --- | --- | --- | --- | --- | --- | --- | --- |
| GO:0031225 | anchored component of membrane | Cellular component | 7 | 159 | 182 | 18698 | 4.5229801644896 | 0.000950112131331274 | 0.767836796174242 | 3.02222513666414 | 0.0384615384615385 | GPC1//RAB3B//LYPD6//EFNA3//NCAM1//SPRN//CNTN2// |
| GO:0098796 | membrane protein complex | Cellular component | 21 | 1166 | 182 | 18698 | 1.8503100672912 | 0.00477131875342303 | 0.767836796174242 | 2.3213615689308 | 0.115384615384615 | PYURF//VPS37D//TOMM40//CPLX1//GOSR2//FAF2//EMC10//EVC//GNAO1//GNAZ//SCN3B//ATP1B4//KCNIP3//CNTN2//CDH7//NDUFA4//GRIN2B//CNIH2//SESTD1//BEST1//NPNT// |
| GO:0043005 | neuron projection | Cellular component | 21 | 1224 | 182 | 18698 | 1.76263197586727 | 0.00816194057480438 | 0.767836796174242 | 2.0882065715486 | 0.115384615384615 | BDNF//BSN//CPLX1//CHRM1//CNIH2//GNAO1//GNAZ//KCNIP3//PURA//MLPH//UNC5A//APBB1//ABHD17B//SPTBN5//FAM161A//DYNLL1//CNTN2//LYPD6//GRIN2B//NPTX1//CAMK2A// |
| GO:0042995 | cell projection | Cellular component | 31 | 2055 | 182 | 18698 | 1.54979278628914 | 0.00885217685327978 | 0.767836796174242 | 2.05294991810263 | 0.17032967032967 | ARFIP2//EVC//TULP4//FAM161A//DYNLL1//LYPD6//GRIN2B//NPTX1//CAMK2A//FAM89B//APBB1//BRK1//ARHGEF6//BDNF//BSN//CPLX1//CHRM1//CNIH2//GNAO1//GNAZ//KCNIP3//PURA//MLPH//ABHD17B//TWF1//UNC5A//KIF2A//SPTBN5//CNTN2//ARHGAP35//FERMT3// |
| GO:0005654 | nucleoplasm | Cellular component | 47 | 3472 | 182 | 18698 | 1.3907270724667 | 0.0093304148569331 | 0.767836796174242 | 2.03009904584929 | 0.258241758241758 | HDAC11//EP300//OGT//ATXN7L3//POLR2E//POLR3H//BAHD1//STK35//MCIDAS//KIF2A//SH3BP5//TP53INP2//APBB1//ARL6IP4//PRKAA1//BDNF//LHPP//SRSF3//KMT2D//CDKN2D//RPP14//NUDT16//FOXO1//PLA2G15//PTPN18//HEYL//GPC1//HOXC9//LMO2//MIF//FOXP3//CTDSPL2//ARID4B//SHQ1//THUMPD1//ASF1B//PSMB8//COPS7B//SRF//PAX8//NUP214//CAMK2A//CASP7//CREB3L3//HAT1//BTAF1//FAM131B// |
| GO:0120025 | plasma membrane bounded cell projection | Cellular component | 30 | 1980 | 182 | 18698 | 1.55661005661006 | 0.00945200368074081 | 0.767836796174242 | 2.02447611791936 | 0.164835164835165 | ARFIP2//EVC//TULP4//FAM161A//DYNLL1//LYPD6//GRIN2B//NPTX1//CAMK2A//FAM89B//APBB1//BRK1//ARHGEF6//BDNF//BSN//CPLX1//CHRM1//CNIH2//GNAO1//GNAZ//KCNIP3//PURA//MLPH//TWF1//ABHD17B//UNC5A//KIF2A//SPTBN5//CNTN2//ARHGAP35// |
| GO:0030425 | dendrite | Cellular component | 12 | 569 | 182 | 18698 | 2.16666988547481 | 0.00997199201291627 | 0.767836796174242 | 2.00121807801702 | 0.0659340659340659 | CNIH2//APBB1//ABHD17B//CPLX1//CHRM1//GNAO1//GNAZ//KCNIP3//PURA//BDNF//MLPH//BSN// |
| GO:0097447 | dendritic tree | Cellular component | 12 | 570 | 182 | 18698 | 2.16286871023713 | 0.0101020487820542 | 0.767836796174242 | 1.99559053864234 | 0.0659340659340659 | CPLX1//CHRM1//CNIH2//GNAO1//GNAZ//KCNIP3//PURA//BDNF//MLPH//BSN//APBB1//ABHD17B// |
| GO:0044428 | nuclear part | Cellular component | 57 | 4435 | 182 | 18698 | 1.32039842907938 | 0.0113807344023825 | 0.767836796174242 | 1.9438297118767 | 0.313186813186813 | ATP1B4//GNAZ//CDKN2D//RPP14//NUDT16//POLR3H//EP300//BAHD1//FOXO1//PLA2G15//PTPN18//HEYL//GPC1//APBB1//HOXC9//LMO2//MIF//FOXP3//CTDSPL2//ARID4B//POLR2E//SHQ1//PRKAA1//THUMPD1//ASF1B//PSMB8//SRSF3//COPS7B//SRF//PAX8//NUP214//KMT2D//CAMK2A//CASP7//CREB3L3//OGT//HAT1//BTAF1//SH3BP5//FAM131B//STK35//KIF2A//SPRN//ARL6IP4//NOL4//PPP1R26//DYNLL1//VAPA//HDAC11//MCIDAS//ATXN7L3//ZNF385B//PURA//TP53INP2//BDNF//LHPP//P2RX2// |
| GO:0005666 | DNA-directed RNA polymerase III complex | Cellular component | 2 | 17 | 182 | 18698 | 12.0866192630899 | 0.0116413119041605 | 0.767836796174242 | 1.93399807461459 | 0.010989010989011 | POLR3H//POLR2E// |
| GO:0098793 | presynapse | Cellular component | 9 | 379 | 182 | 18698 | 2.43964742381629 | 0.0121231455594953 | 0.767836796174242 | 1.91638468034407 | 0.0494505494505494 | RAB3B//BDNF//RIMS4//APBB1//CAMK2A//CPLX1//CHRM1//KCNIP3//BSN// |
| GO:0044451 | nucleoplasm part | Cellular component | 19 | 1113 | 182 | 18698 | 1.75380863521025 | 0.0122879362483023 | 0.767836796174242 | 1.91052105049309 | 0.104395604395604 | HDAC11//EP300//OGT//POLR2E//POLR3H//STK35//MCIDAS//KIF2A//SH3BP5//KMT2D//ATXN7L3//TP53INP2//APBB1//ARL6IP4//PRKAA1//BDNF//LHPP//SRSF3//BAHD1// |
| GO:1902495 | transmembrane transporter complex | Cellular component | 8 | 321 | 182 | 18698 | 2.56040532676047 | 0.0135267202055064 | 0.767836796174242 | 1.86880749307877 | 0.043956043956044 | BEST1//GRIN2B//CNIH2//SESTD1//SCN3B//KCNIP3//CNTN2//ATP1B4// |
| GO:1990351 | transporter complex | Cellular component | 8 | 329 | 182 | 18698 | 2.49814623066903 | 0.0154731819032469 | 0.767836796174242 | 1.8104203688522 | 0.043956043956044 | GRIN2B//CNIH2//SESTD1//BEST1//ATP1B4//SCN3B//KCNIP3//CNTN2// |
| GO:0036477 | somatodendritic compartment | Cellular component | 14 | 766 | 182 | 18698 | 1.87768628238602 | 0.0178399153188935 | 0.767836796174242 | 1.74860721142988 | 0.0769230769230769 | CPLX1//APBB1//PURA//CNTN2//UNC5A//CHRM1//CNIH2//GNAO1//GNAZ//KCNIP3//BDNF//MLPH//BSN//ABHD17B// |
| GO:0031981 | nuclear lumen | Cellular component | 52 | 4071 | 182 | 18698 | 1.31227848545461 | 0.0182294076880672 | 0.767836796174242 | 1.73922744226017 | 0.285714285714286 | CDKN2D//RPP14//NUDT16//POLR3H//EP300//BAHD1//FOXO1//PLA2G15//PTPN18//HEYL//GPC1//APBB1//HOXC9//LMO2//MIF//FOXP3//CTDSPL2//ARID4B//POLR2E//SHQ1//PRKAA1//THUMPD1//ASF1B//PSMB8//SRSF3//COPS7B//SRF//PAX8//NUP214//KMT2D//CAMK2A//CASP7//CREB3L3//OGT//HAT1//BTAF1//SH3BP5//FAM131B//STK35//KIF2A//SPRN//ARL6IP4//NOL4//PPP1R26//PURA//HDAC11//ATXN7L3//MCIDAS//TP53INP2//BDNF//LHPP//ZNF385B// |
| GO:0030863 | cortical cytoskeleton | Cellular component | 4 | 105 | 182 | 18698 | 3.91376242804814 | 0.0194021535385394 | 0.767836796174242 | 1.71215006295651 | 0.021978021978022 | MLPH//RIMS4//BSN//SPTBN5// |
| GO:0034703 | cation channel complex | Cellular component | 6 | 217 | 182 | 18698 | 2.84063402035752 | 0.0196689259376912 | 0.767836796174242 | 1.70621935497999 | 0.032967032967033 | GRIN2B//CNIH2//SESTD1//KCNIP3//CNTN2//SCN3B// |
| GO:0032991 | protein-containing complex | Cellular component | 62 | 5071 | 182 | 18698 | 1.25609314763328 | 0.0227483006174434 | 0.767836796174242 | 1.64305104120613 | 0.340659340659341 | DYNLL1//FERMT3//COL6A6//PURA//EP300//LMO2//PSMB8//COPS7B//PFDN1//CGNL1//YWHAB//TOB1//NUP214//BRK1//KCNIP3//ARMC8//P2RX2//IL4R//ATXN7L3//KIF2A//VPS53//VPS37D//PYURF//TOMM40//CPLX1//GOSR2//FAF2//EMC10//EVC//HDAC11//NPNT//BAHD1//RPP14//NDUFA4//CARHSP1//MEX3A//GNAO1//GNAZ//SCN3B//ATP1B4//CNTN2//CDH7//UBE4B//GPD1L//BEST1//KMT2D//ABCA1//ZSWIM6//GRIN2B//CNIH2//POLR3H//POLR2E//OGT//SESTD1//PRKAA1//CDKN2D//AKAP10//NAT9//FOXP3//FAM86C1//ASF1B//HAT1// |
| GO:0014069 | postsynaptic density | Cellular component | 6 | 227 | 182 | 18698 | 2.71549595778671 | 0.0239074447261845 | 0.767836796174242 | 1.62146683963585 | 0.032967032967033 | GRIN2B//CHRM1//CNIH2//ABHD17B//CAMK2A//BSN// |
| GO:0099572 | postsynaptic specialization | Cellular component | 6 | 229 | 182 | 18698 | 2.69177983588464 | 0.0248231146344203 | 0.767836796174242 | 1.60514372712107 | 0.032967032967033 | CHRM1//CNIH2//GRIN2B//ABHD17B//CAMK2A//BSN// |
| GO:0097458 | neuron part | Cellular component | 23 | 1533 | 182 | 18698 | 1.54137903844362 | 0.0249073712527938 | 0.767836796174242 | 1.6036721058898 | 0.126373626373626 | FAM161A//RAB3B//BDNF//CHRM1//CNIH2//GRIN2B//ABHD17B//CAMK2A//BSN//LYPD6//NPTX1//SPTBN5//UNC5A//RIMS4//APBB1//CPLX1//PURA//CNTN2//GNAO1//GNAZ//KCNIP3//MLPH//DYNLL1// |
| GO:0098797 | plasma membrane protein complex | Cellular component | 11 | 574 | 182 | 18698 | 1.96881341654861 | 0.0251264104026311 | 0.767836796174242 | 1.59986955094904 | 0.0604395604395604 | GNAO1//GNAZ//SCN3B//ATP1B4//KCNIP3//CNTN2//CDH7//NPNT//GRIN2B//CNIH2//EVC// |
| GO:0045202 | synapse | Cellular component | 15 | 880 | 182 | 18698 | 1.75118631368631 | 0.0252214278120874 | 0.767836796174242 | 1.59823033119044 | 0.0824175824175824 | BSN//CHRM1//CNIH2//GRIN2B//ABHD17B//CAMK2A//RAB3B//BDNF//RIMS4//APBB1//CPLX1//KCNIP3//P2RX2//LYPD6//CNTN2// |
| GO:0034702 | ion channel complex | Cellular component | 7 | 296 | 182 | 18698 | 2.4295738045738 | 0.0261064592482631 | 0.767836796174242 | 1.58325202642353 | 0.0384615384615385 | BEST1//GRIN2B//CNIH2//SESTD1//SCN3B//KCNIP3//CNTN2// |
| GO:0032279 | asymmetric synapse | Cellular component | 6 | 232 | 182 | 18698 | 2.65697233800682 | 0.0262401517408681 | 0.767836796174242 | 1.58103365786226 | 0.032967032967033 | CHRM1//CNIH2//GRIN2B//ABHD17B//CAMK2A//BSN// |
| GO:0044463 | cell projection part | Cellular component | 21 | 1377 | 182 | 18698 | 1.56678397854868 | 0.0270328268613483 | 0.767836796174242 | 1.56810853713177 | 0.115384615384615 | CPLX1//CHRM1//CNIH2//GNAO1//GNAZ//KCNIP3//PURA//BDNF//MLPH//BSN//APBB1//ABHD17B//TWF1//UNC5A//EVC//SPTBN5//DYNLL1//CNTN2//FAM161A//ARHGAP35//KIF2A// |
| GO:0120038 | plasma membrane bounded cell projection part | Cellular component | 21 | 1377 | 182 | 18698 | 1.56678397854868 | 0.0270328268613483 | 0.767836796174242 | 1.56810853713177 | 0.115384615384615 | CPLX1//CHRM1//CNIH2//GNAO1//GNAZ//KCNIP3//PURA//BDNF//MLPH//BSN//APBB1//ABHD17B//TWF1//UNC5A//EVC//CNTN2//DYNLL1//ARHGAP35//FAM161A//KIF2A//SPTBN5// |
| GO:0044425 | membrane part | Cellular component | 79 | 6780 | 182 | 18698 | 1.19707445946384 | 0.0273370548231781 | 0.767836796174242 | 1.56324827626931 | 0.434065934065934 | OSR1//PYURF//CSPG5//ABCA1//CNIH2//CYP4F3//MOGAT2//CREB3L3//CYP4F2//VAPA//GOSR2//SH3BP4//CDH7//TOMM40//SEMA4B//SLC26A1//PTPRT//SMIM12//TM4SF18//TMEM132E//TEDDM1//RNF215//EVC//P2RX2//AVL9//TMIE//ZDHHC22//EMC10//ST6GALNAC6//GPR153//LDLRAD2//ADAM11//NCAM1//GPX8//CRIM1//FGFRL1//SCN3B//SEMA4G//ANO3//SFRP1//SLC35E2B//BEST1//TMEM62//TMEM246//UNC5A//B4GALT5//MFAP3L//LYPD6//EFNA3//SPRN//CNTN2//PRKCQ//VPS37D//CPLX1//FAF2//CHRM1//ATP1B4//GPC1//GPR3//GRIN2B//SLC39A2//IL4R//TRABD2B//CLEC1A//NUCB1//RAB3B//NDUFA4//TWF1//GNAO1//GNAZ//KCNIP3//PRKAA1//RIMS4//APBB1//CAMK2A//ABHD17B//NPNT//SPTBN5//SESTD1// |
| GO:0005829 | cytosol | Cellular component | 61 | 5022 | 182 | 18698 | 1.24789169412825 | 0.0273452312783567 | 0.767836796174242 | 1.56311839913193 | 0.335164835164835 | CDKN2D//TOMM40//CPLX1//RALBP1//AKAP10//OSR1//UBXN2B//POLR3H//DPYS//DENND6A//EP300//FOXO1//GPD1L//MGRN1//ISCU//DDAH1//CARHSP1//ARFIP2//SULT4A1//PTPN18//GNAZ//GPC1//ARHGAP35//KCNIP3//KIF2A//MIF//NCAM1//SPRN//SPTBN5//POLR2E//RHOF//SHQ1//VPS53//PRKAA1//BRK1//PRKCQ//ZNF395//PSMB8//TULP4//TWF1//TP53INP2//PCTP//RAB3B//LHPP//SFRP1//COPS7B//BEST1//YWHAB//BTG2//NUP214//CAMK2A//KRTAP1-1//CASP7//CREB3L3//OGT//DYNLL1//MEX3A//MYL10//ARHGEF6//GOSR2//FAM131B// |
| GO:0098984 | neuron to neuron synapse | Cellular component | 6 | 235 | 182 | 18698 | 2.62305354220248 | 0.0277100590004674 | 0.767836796174242 | 1.55736254951369 | 0.032967032967033 | CHRM1//CNIH2//GRIN2B//ABHD17B//CAMK2A//BSN// |
| GO:0044456 | synapse part | Cellular component | 13 | 740 | 182 | 18698 | 1.80482625482625 | 0.0291887505630006 | 0.783535522925547 | 1.53478449477587 | 0.0714285714285714 | RAB3B//BDNF//RIMS4//BSN//P2RX2//APBB1//CAMK2A//CHRM1//CNIH2//GRIN2B//ABHD17B//CPLX1//KCNIP3// |
| GO:1902494 | catalytic complex | Cellular component | 21 | 1417 | 182 | 18698 | 1.5225557787308 | 0.0353210608799561 | 0.833097013206293 | 1.45196626071758 | 0.115384615384615 | HDAC11//DYNLL1//GNAO1//GNAZ//ATXN7L3//NDUFA4//GPD1L//UBE4B//PYURF//ZSWIM6//CARHSP1//KMT2D//POLR3H//POLR2E//PRKAA1//ATP1B4//EP300//OGT//RPP14//PSMB8//CDKN2D// |
| GO:0031974 | membrane-enclosed lumen | Cellular component | 62 | 5188 | 182 | 18698 | 1.22776568073407 | 0.0355072574539817 | 0.833097013206293 | 1.44968287088621 | 0.340659340659341 | MIF//ISCU//ENAM//NUCB1//GPX8//CSPG5//GPC1//ARMC8//FAF2//SMPD1//ISLR//FERMT3//CDKN2D//RPP14//NUDT16//POLR3H//EP300//BAHD1//FOXO1//PLA2G15//PTPN18//HEYL//APBB1//HOXC9//LMO2//FOXP3//CTDSPL2//ARID4B//POLR2E//SHQ1//PRKAA1//THUMPD1//ASF1B//PSMB8//SRSF3//COPS7B//SRF//PAX8//NUP214//KMT2D//CAMK2A//CASP7//CREB3L3//OGT//HAT1//BTAF1//SH3BP5//FAM131B//STK35//KIF2A//SPRN//ARL6IP4//NOL4//PPP1R26//PURA//HDAC11//ATXN7L3//MCIDAS//TP53INP2//BDNF//LHPP//ZNF385B// |
| GO:0043233 | organelle lumen | Cellular component | 62 | 5188 | 182 | 18698 | 1.22776568073407 | 0.0355072574539817 | 0.833097013206293 | 1.44968287088621 | 0.340659340659341 | ISCU//ENAM//NUCB1//GPX8//CSPG5//GPC1//ARMC8//MIF//FAF2//SMPD1//ISLR//FERMT3//CDKN2D//RPP14//NUDT16//POLR3H//EP300//BAHD1//FOXO1//PLA2G15//PTPN18//HEYL//APBB1//HOXC9//LMO2//FOXP3//CTDSPL2//ARID4B//POLR2E//SHQ1//PRKAA1//THUMPD1//ASF1B//PSMB8//SRSF3//COPS7B//SRF//PAX8//NUP214//KMT2D//CAMK2A//CASP7//CREB3L3//OGT//HAT1//BTAF1//SH3BP5//FAM131B//STK35//KIF2A//SPRN//ARL6IP4//NOL4//PPP1R26//PURA//HDAC11//ATXN7L3//MCIDAS//TP53INP2//BDNF//LHPP//ZNF385B// |
| GO:0070013 | intracellular organelle lumen | Cellular component | 62 | 5188 | 182 | 18698 | 1.22776568073407 | 0.0355072574539817 | 0.833097013206293 | 1.44968287088621 | 0.340659340659341 | ISCU//ENAM//NUCB1//GPX8//CSPG5//GPC1//ARMC8//MIF//FAF2//SMPD1//ISLR//FERMT3//CDKN2D//RPP14//NUDT16//POLR3H//EP300//BAHD1//FOXO1//PLA2G15//PTPN18//HEYL//APBB1//HOXC9//LMO2//FOXP3//CTDSPL2//ARID4B//POLR2E//SHQ1//PRKAA1//THUMPD1//ASF1B//PSMB8//SRSF3//COPS7B//SRF//PAX8//NUP214//KMT2D//CAMK2A//CASP7//CREB3L3//OGT//HAT1//BTAF1//SH3BP5//FAM131B//STK35//KIF2A//SPRN//ARL6IP4//NOL4//PPP1R26//PURA//HDAC11//ATXN7L3//MCIDAS//TP53INP2//BDNF//LHPP//ZNF385B// |
| GO:0097060 | synaptic membrane | Cellular component | 7 | 317 | 182 | 18698 | 2.26862412035914 | 0.0358842718144736 | 0.833097013206293 | 1.44509586231071 | 0.0384615384615385 | RIMS4//APBB1//CAMK2A//CHRM1//CNIH2//GRIN2B//ABHD17B// |
| GO:0042734 | presynaptic membrane | Cellular component | 3 | 79 | 182 | 18698 | 3.90137710390875 | 0.041881262822525 | 0.912957791295674 | 1.37798023175476 | 0.0164835164835165 | RIMS4//APBB1//CAMK2A// |
| GO:0099738 | cell cortex region | Cellular component | 2 | 34 | 182 | 18698 | 6.04330963154493 | 0.0431262868576772 | 0.912957791295674 | 1.36525793268737 | 0.010989010989011 | RIMS4//BSN// |
| GO:0048786 | presynaptic active zone | Cellular component | 2 | 35 | 182 | 18698 | 5.87064364207221 | 0.0454537993723995 | 0.912957791295674 | 1.34242980930661 | 0.010989010989011 | RIMS4//BSN// |
| GO:0044306 | neuron projection terminus | Cellular component | 4 | 139 | 182 | 18698 | 2.95643924420903 | 0.0470527361147529 | 0.912957791295674 | 1.3274151172954 | 0.021978021978022 | CHRM1//KCNIP3//CPLX1//BSN// |
| GO:0000123 | histone acetyltransferase complex | Cellular component | 3 | 83 | 182 | 18698 | 3.7133589302264 | 0.0473287622632957 | 0.912957791295674 | 1.32487485300611 | 0.0164835164835165 | ATXN7L3//EP300//OGT// |
| GO:0005834 | heterotrimeric G-protein complex | Cellular component | 2 | 36 | 182 | 18698 | 5.70757020757021 | 0.0478266596138595 | 0.912957791295674 | 1.32032995075941 | 0.010989010989011 | GNAO1//GNAZ// |
| GO:0032391 | photoreceptor connecting cilium | Cellular component | 2 | 36 | 182 | 18698 | 5.70757020757021 | 0.0478266596138595 | 0.912957791295674 | 1.32032995075941 | 0.010989010989011 | SPTBN5//FAM161A// |
| GO:1905360 | GTPase complex | Cellular component | 2 | 36 | 182 | 18698 | 5.70757020757021 | 0.0478266596138595 | 0.912957791295674 | 1.32032995075941 | 0.010989010989011 | GNAO1//GNAZ// |
